# Translating Discoveries in Attention-Deficit/Hyperactivity Disorder Genomics to an Outpatient Child and Adolescent Psychiatric Cohort

**DOI:** 10.1016/j.jaac.2019.08.004

**Published:** 2020-08

**Authors:** Pieter J. Vuijk, Joanna Martin, Ellen B. Braaten, Giulio Genovese, Michael R. Capawana, Sheila M. O’Keefe, B. Andi Lee, Hannah S. Lind, Jordan W. Smoller, Stephen V. Faraone, Roy H. Perlis, Alysa E. Doyle

**Affiliations:** a Center for Genomic Medicine, Massachusetts General Hospital, Boston; b MRC Centre for Neuropsychiatric Genetics and Genomics, Cardiff University, UK; c Stanley Center for Psychiatric Research, Broad Institute, Cambridge, MA; d Massachusetts General Hospital and Harvard Medical School, Massachusetts General Hospital, Boston; e SUNY Upstate Medical University, Syracuse, NY; f Center for Experimental Drugs and Diagnostics, Massachusetts General Hospital, Boston

**Keywords:** ADHD, clinical translation, genomic medicine, polygenic risk

## Abstract

**Objective:**

Genomic discoveries should be investigated in generalizable child psychiatric samples in order to justify and inform studies that will evaluate their use for specific clinical purposes. In youth consecutively referred for neuropsychiatric evaluation, we examined 1) the convergent and discriminant validity of attention-deficit/hyperactivity disorder (ADHD) polygenic risk scores (PRSs) in relation to *DSM*-based ADHD phenotypes; 2) the association of ADHD PRSs with phenotypes beyond ADHD that share its liability and have implications for outcome; and 3) the extent to which youth with high ADHD PRSs manifest a distinctive clinical profile.

**Method:**

Participants were 433 youth, ages 7–18 years, from the Longitudinal Study of Genetic Influences on Cognition. We used logistic/linear regression and mixed effects models to examine associations with ADHD-related polygenic variation from the largest ADHD genome-wide association study to date. We replicated key findings in 5,140 adult patients from a local health system biobank.

**Results:**

Among referred youth, ADHD PRSs were associated with ADHD diagnoses, cross-diagnostic ADHD symptoms and academic impairment (odds ratios ∼1.4; *R*^*2*^ values ∼2%–3%), as well as cross-diagnostic variation in aggression and working memory. In adults, ADHD PRSs were associated with ADHD and phenotypes beyond the condition that have public health implications. Finally, youth with a high ADHD polygenic burden showed a more severe clinical profile than youth with a low burden (β coefficients ∼.2).

**Conclusion:**

Among child and adolescent outpatients, ADHD polygenic risk was associated with ADHD and related phenotypes as well as clinical severity. These results extend the scientific foundation for studies of ADHD polygenic risk in the clinical setting and highlight directions for further research.

Given progress in the identification of genetic variants that increase risk for neuropsychiatric illness,^1^ determining ways to leverage these discoveries in the clinical setting is now a priority for the field of psychiatric genetics.^2^ For child psychiatry, this issue is timely. Attention-deficit/hyperactivity disorder (ADHD) is one of the most common child-onset conditions.^3^ Although twin studies^4^ have long supported its heritability, a recent genome-wide association study (GWAS)^5^ has, for the first time, implicated specific common variants in its susceptibility, using stringent criteria for significance and replication. This groundbreaking analysis^5^ revealed 12 genome-wide significant loci, several of which highlight molecular processes not previously considered for ADHD etiology. Such variants represent the tail of a polygenic component, identified in prior studies^6^, ^7^ and now refined, which reflects the aggregate influence of potentially thousands of small-effect alleles.

Although the pathophysiology underlying these discoveries is not resolved, this polygenic signal is itself of clinical interest as an objective index of ADHD susceptibility. Given a lack of biomarkers, child psychiatric diagnoses are based on caregiver and teacher ratings and behavioral observations. While these information sources show construct validity,^8^ reports can vary because of setting-specific behaviors and discrepant standards regarding age-appropriate variation. Thus, even for experienced clinicians, there can be tension between making an efficient diagnosis and guarding against overmedicating and overlabeling children. Furthermore, ADHD is associated with a range of functional outcomes,^3^ and childhood symptom tallies are not useful prognostic indicators.^9^ Given the complex genetic architecture of ADHD, the shared liability between ADHD and other neuropsychiatric conditions,^10^ and the probabilistic role of susceptibility variants, polygenic risk scores (PRSs) are unlikely to yield large magnitude improvements in diagnostic efficiency or risk stratification. Yet, in the absence of other objective indicators, even modest relationships to psychopathology and/or clinical severity could have value.

Practically, moving from genetic discoveries to patient care requires intermediate phases of clinical research. As Green *et al.*^11^ note, studies that augment clinical scientific knowledge are often needed before studies that evaluate the deployment of genomic information in the clinic, although these types of clinical studies ultimately become iterative. For ADHD PRSs, gaps in clinical science require consideration. PRSs based on ADHD GWAS discovery samples have been related to ADHD diagnosis, dimensional ADHD symptoms, and learning/educational impairment in independent case-control and population-based cohorts (Table 1). Yet, because ADHD shares liability with other neuropsychiatric conditions,^10^ whether ADHD PRSs would show convergent validity with ADHD-related phenotypes among referred youths remains an open question. Additionally, to have clinical value, ADHD PRS→phenotype associations need not segregate along DSM boundaries, as long as relationships are reliable. Indeed, as anticipated by the National Institute of Mental Health Research Domain Criteria framework,^12^ genomic discoveries may promote a more biologically informed psychiatric nosology. As also shown in Table 1, population and research samples have related ADHD PRSs to both cognitive and behavioral phenotypes beyond the conditions that putatively share its liability. Among these, associations with aggression^6^ and working memory (WM)^13^ are important to confirm in the outpatient setting because of their implications for outcome.^14^, ^15^, ^16^, ^17^

**Table 1:** Published Studies Examining the Association Between Attention-Deficit/Hyperactivity Disorder (ADHD) Polygenic Risk and ADHD or Phenotypes Beyond ADHD

Study	Sample Type (N)	Discovery Sample for ADHD Risk Score (N)	Phenotypes Associated With ADHD Polygenic Risk		Phenotypes Not Associated With ADHD Polygenic Risk	Additional Details, Implications, and Comments
			ADHD-Related Phenotypes	Phenotypes Beyond ADHD		
Hamshere *et al.*, 2013^6^	Case-control design (n = 452 ADHD vs. n = 5,081 controls)	Neale *et al.*, 2010^54^ (N = 2,064 trios, 896 cases, 2,455 controls)	ADHD dx	CD dx and sx; aggression sx	N/A	ADHD PRSs were elevated in ADHD cases vs. comparisons, higher in ADHD+CD vs. ADHD without CD, and associated with comorbid CD (particularly aggression) sx.
Groen-Blokhuis *et al.*, 2014^49^	Population sample (Netherlands Twin Register; N = 2,437)	Holmans, 2013^55^—WCPG (n = 5,621 ADHD cases, n = 13,589 controls)	Maternal rated ADHD sx at preschool and school age; teacher-rated ADHD sx at school age	N/A	N/A	ADHD PRSs were associated with dimensional ADHD sx across different raters and at 2 developmental periods.
Martin *et al.*, 2014^7^	Population sample (ALSPAC; N = 5,661)	UK/Irish ADHD GWAS (n = 727 cases and n = 5,081 controls)	Parent-rated ADHD sx; INATT and HYP/IMP sx	PL	SC	ADHD PRSs were most strongly associated with total ADHD symptoms and HYP/IMP sx but also associated with INATT sx. PRSs were also associated with 1 of 2 autism-related traits.
Martin *et al.*, 2015^13^	Population sample (ALSPAC; N = 6,832)	UK/Irish ADHD GWAS (n = 727 cases and n = 5,081 controls)	Parent-rated ADHD sx	Latent neurodevelopmental factor (including ADHD sx and PL), IQ, WM	Inhibitory control; emotion recognition	ADHD PRSs were associated with IQ and WM above and beyond associations with latent neurodevelopmental factor using structural equation modeling. Association with IQ was replicated using a second discovery sample.
Stergiakouli *et al.*, 2015^50^	Case-control sample (n = 508 vs. n = 5,081)	Derived from ALSPAC (N = 4,546)	ADHD case status; total ADHD sx and INATT sx	N/A	Trend-level association for HYP/IMP sx in expected direction	ADHD PRSs distinguished ADHD dx, ADHD sx severity but stronger association with INATT sx than HYP/IMP sx (opposite pattern to that in Martin *et al.*, 2014^7^).
Riglin *et al.*, 2016^9^	Population sample (ALSPAC; N = 9,757)	Neale *et al.*, 2010^54^ (N = 2,064 trios, 896 cases, 2,455 controls)	Trajectory of ADHD sx	ADHD-related neurodevelopmental and conduct problems thought to share liability with ADHD (called multimorbidity; comprising IQ <80, SC, PL, conduct problems)	N/A	Examined 4 ADHD sx trajectories (low, intermediate, child-limited, and persistent). ADHD PRSs were higher in persistent group vs. other 3). Multimorbidity was higher in persistent trajectory and associated with persistence independent of ADHD PRSs. After controlling for multimorbidity, ADHD PRSs were not associated with sx trajectories/persistence. Risk scores from SCZ, BPD, and MDD were not associated with ADHD trajectories.
Benca *et al.*, 2017^30^	Unrelated individuals from population-based Colorado twin study (N = 386)	Neale *et al.*, 2010^54^ (N = 2,064 trios, 896 cases, 2,455 controls)	N/A	N/A	ADHD sx; ADHD dx; 3 latent EF variables (common—variance from EF tasks; variance specific to updating and shifting)	No robust findings. Analyses likely underpowered.
Carey *et al.*, 2017^51^	College students in Duke Neurogenetics study (N = 404)	ADHD cases from PGC cross-disorder analysis (N = 1,947 trio cases, 1,947 trio pseudocontrols, 840 cases, 688 controls)	N/A	VS activity	Self-reported PA	ADHD PRSs were associated with bilateral VS activity. ASD PRSs used as controls; were not associated with VS activity. ADHD PRSs were not directly associated with PA, but VS activity was associated. Path model suggested indirect influence of ADHD PRSs on PA through VS activity.
Riglin *et al.*, 2017^52^	Two population samples (ALSPAC and NCDS); 1 ADHD clinical sample (SAGE; N = 569)	Nine ADHD studies in ALSPAC analysis (n = 5,621 cases, n = 13,589 controls); SAGE and NCDS samples removed for their analyses	N/A	Parent-reported irritability	N/A	ADHD PRSs were associated with irritability in ALSPAC at different ages and in ADHD clinical sample (higher OR). ADHD sx also associated with irritability. No evidence of age or sex differences. MDD PRSs were not associated with irritability.
Stergiakouli *et al.*, 2017^39^	Population-based (ALSPAC; n = 8,365 children and n = 8,340 mothers)	UK/Irish ADHD GWAS (n = 727 ADHD cases and n = 5,081 controls)	N/A	Academic performance/educational outcomes and IQ	N/A	In youths and mothers, ADHD PRSs were associated with educational outcomes and IQ. In youths, influence of ADHD PRSs on educational outcomes was mediated substantially by IQ and partially by ADHD sx.
Du Rietz *et al.*, 2017^53^	UK biobank; adult community/population sample 40–73 (N = 135,726)	PGC-iPSYCH ADHD meta-analysis (n = 20,183 cases and n = 35,191 controls)^5^	N/A	ADHD-related: BMI, neuroticism, DEP, ANX (suggestive), risk taking, ALC (intake and DEP), smoking, V-N reasoning, neuroticism items (including irritability and mood swings); control traits: age	SCZ or BPD. Seven of eight control traits (height, year of assessment, menstruation during assessment, grip strength, visual acuity, self-reported cancers, sex of baby)	ADHD PRSs were associated with a range of conditions likely related to ADHD liability; controls for dx group analyses were individuals with no dx. Could not address ADHD (or ODD, CD, ASD) because of low rates in sample. No sex-specific effects. Seven of eight control traits showed no association.
Nigg *et al.*, 2018^40^	Case-control community volunteer children aged 7–11 (n = 656; primary model n = 337 ADHD and 177 non-ADHD)	PGC-iPSYCH ADHD meta-analysis (n = 20,183 cases and n = 35,191 controls)^5^	ADHD dx; parent and teacher ADHD sx	Two of 5 latent cognitive constructs (WM and V/A)	Three of 5 latent cognitive constructs (trend-level associations with MC and PS; no association with INHIB)	ADHD PRSs were associated with ADHD dx, parent- and teacher-rated dimensional sx, WM and V/A, after medication washout. In models, WM and V/A partially mediated association between WM and V/A and ADHD (dx and dimensions).
Brikell *et al.*, 2018^38^	N = 13,457 children aged either 9 or 12 from CATSS	PGC-iPSYCH ADHD meta-analysis (n = 20,183 cases and n = 35,191 controls)^5^	Parent-rated ADHD sx	Parent-rated neurodevelopmental, externalizing and depression sx	Parent-rated anxiety sx	ADHD PRSs were associated with ADHD sx, as well as neurodevelopmental, externalizing, and depression sx. Associations were largely accounted for by a general childhood psychopathology factor; however, results also showed that ADHD PRSs had an additional, unique association with HYP/IMP sx.

Note: ALC = alcohol; ALSPAC = Avon Longitudinal Study of Parents and Children; ANX = anxiety disorder; ASD = autism spectrum disorder; BMI = body mass index; BPD = bipolar disorder; CATSS = Child and Adolescent Twin Study in Sweden; CD = conduct disorder; DEP = depressive disorder; dx = diagnosis; EF = executive functioning; HYP/IMP = hyperactivity/impulsivity; INATT = inattention; INHIB = inhibition; MC = mental clock; MDD = major depressive disorder; N/A = not applicable; NCDS = National Child Development Study; OR = odds ratio; PA = problematic alcohol use; PGC = Psychiatric Genomics Consortium; PL = pragmatic language; PRS = polygenic risk score; PS = processing speed; SAGE = Study of ADHD, Genes and Environment; SC = social cognition; SCZ = schizophrenia; sx = symptoms; V/A = vigilance/arousal; V-N = verbal-numeric; VS = ventral striatum; WCPG = World Congress of Psychiatric Genetics; WM = working memory.

To our knowledge, a generalizable child and adolescent clinical sample suitable for translating emerging genomic discoveries has not been available. Thus, we have initiated the Longitudinal Study of Genetic Influences on Cognition (LOGIC), which ascertains youths consecutively referred for a neuropsychiatric evaluation. In this article, we used this cohort to address the following three questions: 1) Do ADHD PRSs show convergent and discriminant validity with key ADHD phenotypes in referred youths with a range of psychopathology? 2) In such a sample, are ADHD PRSs associated with phenotypes beyond ADHD that share its liability and relate to functional outcome? 3) Do outpatients with a high ADHD polygenic burden show a distinctive clinical profile? We also examined the themes of questions 1 and 2 in adults from the same health care system to provide a conceptual replication of key questions in patients and to extend our inquiry to adulthood. Given prior studies, we expected ADHD PRSs to associate with ADHD and related phenotypes as well as to clinical severity in youth psychiatric outpatients. If so, such data would justify and inform further research leveraging ADHD PRSs as objective risk indicators and tools for risk stratification in the child psychiatric setting.

## Method

### Subject Recruitment

Participants were youth who were referred to a neuropsychiatric assessment clinic and who agreed to enroll in research. The clinic, housed within the Psychiatry Department of an academic hospital, provides evaluations to assist with differential diagnosis and/or treatment/educational planning. The study recruits consecutive referrals before evaluation. To enroll, youth must provide their clinical data. They are also asked to provide a DNA sample and supplemental assessments to create a uniform phenotype battery.

The study is ongoing. Here, we report a planned analysis of the first wave of genotyped youth. We included unrelated subjects who were 7–18 years of age; had been genotyped by the time of the analysis; and were of European ancestry (based on genomic data), in line with current best practice given the composition of the discovery GWAS. We excluded (n = 4) youth with moderate to severe intellectual disability (mental retardation per *DSM-IV*, Full Scale IQ <55). The inclusion criteria were met by 433 unrelated youth. Their mean age ± SD was 11.5 ± 3.1 years, 62.8% were male, and mean Full Scale IQ ± SD was 100.1 ± 14.5 (range, 55–136).

The study was approved by the Partners Healthcare Institutional Review Board. For youths aged 7–17, parents provided written informed consent and youth provided assent. Youth aged 18 provided written consent.

### Phenotypes

#### Psychopathology

Participants received *DSM-IV-TR* Axis I diagnoses by or under the supervision of doctoral-level licensed psychologists who were hospital faculty. See Supplement 1, available online, for details about this process. Diagnoses included the following: ADHD (full n = 255, 58.9%; subthreshold n = 68, 15.7%); autistic disorder (n = 6, 1.4%), Asperger’s syndrome (n = 34, 7.9%), pervasive developmental disorder not otherwise specified (NOS) (n = 33, 7.6%); oppositional defiant or conduct disorder (n = 122, 28.2%); bipolar disorder (n = 12, 2.8%); major depressive disorder (n = 38, 8.8%), dysthymic disorder (n = 2, 0.5%), mood disorder NOS (n = 48, 11.1%); panic disorder (n = 3, 0.7%), obsessive-compulsive disorder (n = 11, 2.5%), generalized anxiety disorder (n = 34, 7.9%), anxiety disorder NOS (n = 96, 22.2%); schizophrenia (SCZ)/schizoaffective disorder (n = 1, 0.2%), psychotic disorder NOS (n = 12, 2.8%); intellectual disability (n = 6, 1.4%). Additionally, 16.9% of referred youths did not meet criteria for a full Axis I diagnosis, despite having symptoms. Rates surpassed 100% owing to comorbidity. Numbers of conditions per patient were 0 (16.9%), 1 (34.6%), 2 (26.1%), and ≥3 (22.4%).

Parent ratings of dimensional psychopathology symptoms were selected a priori for use in subsequent analyses. Measures (with Cronbach α) included the following: Child Behavior Checklist (CBCL)/6–18: Attention Deficit Hyperactivity Problems (.84), Aggressive Behavior (.94), Somatic Complaints (.78), and Anxiety Problems (.72); Child Symptom Inventory (CSI)-IV^18^: Inattention (.92), Hyperactivity/Impulsivity (.92), and Depression (.86); Child Mania Rating Scale (CMRS)^19^: mania (.73) and irritability (.82) symptoms; and Social Responsiveness Scale^20^: Social Communication/ Interaction (.85) and Social Cognition (.80). We used parent ratings because they have been shown to have higher heritability than youth self-reports (which may have lower reliability and thus more error variance).^21^

#### Cognition and Academic Achievement

Children completed psychometric tests, which were administered using published instructions. Full Scale IQ and WM were operationalized via index scores from the Wechsler Intelligence Scale for Children–Fourth Edition^22^ for youth aged 7–16 and the Wechsler Adult Intelligence Scale–Fourth Edition^23^ for youth aged 17–18. Academic achievement was examined using Word Reading and Numerical Operations of the Wechsler Individual Achievement Test–Third Edition (WIAT-III).^24^

### Other Sample Characteristics

No constraints regarding prior treatment were placed on enrollment, with 59.7% of participants having received prior psychotherapy and/or psychotropic medications. Parent reports (Table S1, available online) were used to create a binary medication use variable. Within the sample, 42.7% of participants were taking ≥1 psychotropic medication (23.3% stimulants).

### Genotyping and Polygenic Risk Scores

DNA was collected via blood venipuncture or by Oragene saliva kits (DNA Genotek, Ottawa, Ontario, Canada) and was extracted at the Broad Institute of MIT and Harvard. Using the Infinium PsychChip v1.0 Psych array (Illumina, San Diego, California), we genotyped all youths with DNA available at the time. We used standard filters and quality control procedures (detailed in Supplement 1, available online). Principal component analysis including individuals in the 1000 Genomes Project^25^ was used to exclude individuals of non-European ancestry. Principal component analysis of the remaining individuals was used to derive covariates reflecting residual population stratification. Risk scores were calculated with PLINK^26^ using the most recently available GWAS for ADHD,^5^ SCZ,^27^ and autism spectrum disorder (ASD).^28^ Scores were generated for 12 *p* value thresholds from the discovery samples. The most stringent thresholds include single nucleotide polymorphisms (SNPs) with the strongest relationship to the diagnosis in the discovery meta-analysis, with more relaxed thresholds incorporating increasing numbers of risk-conferring SNPs. PRSs were standardized within the cohort. See Table S2, available online, for numbers of SNPs at each threshold for ADHD, SCZ, and ASD PRSs. For ADHD and ASD, given low numbers of SNPs, PRSs based on the top two and three thresholds, respectively, were not analyzed.

### Analytic Approach: Youth Sample

For ADHD, we examined scores based on the 10 remaining *p* value thresholds, as there is no precedent in the literature for selecting a single threshold in a heterogeneous clinical cohort and because true relationships would likely yield associations across thresholds.^29^ As PRSs from different thresholds were correlated (Table S3, available online), we addressed multiple comparisons in two ways. First, we used permutation testing based on 10,000 randomly shuffled data sets to generate a null distribution of the sample test statistic.^30^ For questions 1 and 2, we also used a Bonferroni-corrected α to correct for multiple outcomes.

#### Questions 1 and 2

Regarding convergent validity, we examined the association between ADHD PRSs and *DSM*-based ADHD phenotypes (diagnoses, ADHD dimensional symptoms, and academic performance in reading and math). We selected the CBCL ADH scale to represent dimensional ADHD symptoms owing to its prior evidence of heritability^31^ and given our interest in a dimensional representation of the ADHD construct overall. To establish discriminant validity, we first related ADHD PRSs to two traits (ie, somatic complaints and social cognition) that were not expected to be associated with ADHD based on factor-analytic phenotype studies^32^ and a prior ADHD PRS analysis,^7^ respectively. We then related PRSs for SCZ and ASD to ADHD status and ADHD symptoms. Regarding associations beyond *DSM*-defined ADHD, we related the ADHD PRS to the CBCL Aggressive Behavior scale and the Wechsler WM index.

We used hierarchical logistic regression for the dichotomous ADHD diagnosis and hierarchical linear regression for dimensional traits. In step 1, we controlled for age, sex, and the first five principal ancestry components. For WM and achievement, we also controlled for medication use, given that medications could impact test performance. Because nonstimulant medications can be prescribed for ADHD and because patients are not always aware of the symptoms their medications are intended to address, we controlled for any medication use. In step 2, we entered the ADHD PRS. For the logistic regression, model significance was determined by Wald’s χ^2^ test, and an odds ratio (OR) reflected the effect size. For the linear regressions, model significance was determined by an *F* test and *R*^*2*^ values, and β coefficients reflect effect sizes. For question 1, our Bonferroni-corrected threshold was .0083 (.05/6 outcomes, reflecting the dichotomous ADHD diagnosis and the five dimensions hypothesized to be associated with risk scores [ie working memory, word reading, numerical operations, ADHD symptoms, and aggression]). Sensitivity analyses were conducted in all significant analyses by determining whether associations remained after controlling for (broad) ADHD.

#### Question 3

We used multivariate mixed modeling to determine whether the profile of scores across key psychopathology domains differed in youths with a low (bottom 30%), medium (middle 40%), and high (top 30%) ADHD polygenic burden. Eight domains (ie, inattention, hyperactivity, aggression, irritability, mania, social communication/interaction, depression, and anxiety) were standardized based on the mean and SD of the current sample. Models sought to determine a main effect for risk group (ie, whether the overall severity of the symptom profile differed based on polygenic burden) as well as an interaction between risk group and psychopathology domain (ie, whether the severity of particular symptom domains differed as a function of polygenic burden).

Mixed effects modeling is an extension of regular regression that is appropriate when data are hierarchically structured (eg, psychopathology scores within individuals). The technique does not require the data to be balanced, presuming data are missing at random.^33^ Although we could not confirm that data were missing completely at random given Little’s MCAR test (χ^2^ (76) = 104.93, *p* = .02), covariate-dependent missingness in relation to age and sex did yield a satisfying test statistic (χ^2^ (228) = 213.75, *p* = .74), indicating that mixed modeling was appropriate with age and sex as covariates.

### Biobank Replication

We used data on patients from the same hospital’s biobank to conceptually replicate and extend our findings. The biobank enrolls adults from the Partners Healthcare system on a continuous basis.^34^ At the time of analysis, genotyping had been completed in three waves using the Multi-Ethnic Global Array (MEGA) (Illumina). Preparation of genomic data, including standard quality control, data cleaning, and ancestry determination, has been described elsewhere.^35^ All available individuals determined to be of European ancestry whose samples passed quality control were eligible (N = 11,075). Given the potential for extreme generational differences in diagnoses, we excluded adults aged >60 years. Our final sample included 5,140 individuals aged 19–60 years. Calculation of ADHD PRSs was based on the same summary statistics as our youth sample (ie, same discovery GWAS^5^ and same 10 *p* value thresholds). Linkage-disequilibrium pruning of the SNP list was done by applying the clump function from PLINK 1.9, with a 250-kb window and a minimum *r*^*2*^ that was set at 0.2.

We obtained diagnoses and demographics from electronic health records. First, we compared patients with ADHD (ICD-10 code F90) with all other patients. Secondary analyses compared patients with ADHD only with patients with mental, behavioral, and neurodevelopmental disorders (ICD-10 codes F1–F98 excluding F90). Regarding educational attainment, we dichotomized adults who had and had not completed college by age 23 years. This age cutoff was used to capture participants who worked or took a gap year between high school and college. Additionally, we examined ADHD PRSs in relation to the presence or absence of a substance use disorder (SUD) history (ICD-10 codes F10–F19), given associations of SUDs with ADHD liability^36^ and public health outcomes.^37^

We used logistic regression to examine whether ADHD PRS was associated with an elevated relative risk for the three outcomes. We controlled for age, sex, the first five ancestry components, and biobank genotyping wave. As in youths, we used permutation testing to generate the null distribution of the test statistic from the sample. We used a Bonferroni-corrected α of .0167 (.05/3 tests). For outcomes other than ADHD, we ran sensitivity analyses controlling for ADHD status.

## Results

### Question 1. Convergent and Discriminant Validity of ADHD PRSs

Among referred youth, multinomial logistic regression of the ADHD diagnosis variable (full, subthreshold, and none) showed that variation in ADHD PRSs distinguished between levels of the diagnosis at eight discovery sample thresholds. These results were driven by significant differences between youth with full diagnoses and no diagnoses of ADHD, whereas only the most stringent threshold differed between full and subthreshold ADHD (Table S4, available online). Therefore, we collapsed full and subthreshold ADHD diagnoses into one category for subsequent analyses. As shown in Table 2, ADHD PRSs were associated with this broad ADHD diagnosis at six discovery sample thresholds after correction for multiple testing, with significant ORs between 1.35 and 1.42. Thus, among referred youths, every increase of 1.0 SD in these PRSs increased the odds of a diagnosis on the ADHD spectrum 1.4-fold.

**Table 2:** Association of Attention-Deficit/Hyperactivity Disorder (ADHD) Polygenic Risk Score (PRS) in Referred Youth With ADHD Diagnosis, Controlling for Age, Sex, and Ancestry Components

Discovery Threshold	ADHD Spectrum (n = 323) vs. All Others in Clinical Cohort (n = 110)				
	OR	95% CI	Pseudo *R*^*2*^	Wald χ^2^_1_	Permuted *p*
***p* < 1.0 × 10**^**−6**^	**1.40**	**1.11–1.77**	**1.73**	**8.22**	**.0032**
***p* < 1.0 × 10**^**−5**^	**1.38**	**1.09–1.74**	**1.52**	**7.23**	**.0050**
*p* < 1.0 × 10^−4^	1.26	1.01–1.58	0.84	4.02	.0397
*p* < .001	1.26	1.01–1.58	0.85	4.06	.0408
***p* < .01**	**1.44**	**1.14–1.81**	**2.01**	**9.43**	**.0011**
*p* < .05	1.31	1.05–1.64	1.15	5.56	.0165
***p* < .1**	**1.35**	**1.08–1.69**	**1.45**	**6.98**	**.0078**
*p* < .3	1.33	1.07–1.67	1.31	6.33	.0119
***p* < .5**	**1.39**	**1.11–1.74**	**1.71**	**8.18**	**.0034**
***p* < 1**	**1.42**	**1.13–1.78**	**1.90**	**9.07**	**.0019**

Note: Boldface type indicates statistically significant findings after correction. Bonferroni corrected critical value = .008. OR = odds ratio; PRS = Polygenic Risk Score.

ADHD PRS was also significantly associated with dimensional ADHD symptoms at the three most stringent discovery sample thresholds (*R*^*2*^ values, 1.84%–2.93%) (Table S5, available online). Secondary analyses of individual ADHD symptom dimensions were conducted using the CSI, as this measure includes scales representing both of the core *DSM* symptom domains of inattention and hyperactivity/impulsivity. Results (Table S5, available online) were consistent with some,^7^, ^38^ but not all,^39^ prior studies (Table 1) that addressed this issue, in that they suggest a relationship with hyperactivity/impulsivity rather than inattention symptoms.

Variation in ADHD PRSs was also associated with lower academic achievement (Table 3). For word reading, four significant associations were found at inclusive *p* value thresholds (*R*^*2*^ values, 1.88%–2.05%). For numerical operations, three significant associations were found (*R*^*2*^ values, 1.94%–2.27%). Controlling for ADHD did not reduce the number of significant findings for reading, and one significant threshold remained for math achievement (Table S6, available online). Controlling for stimulants instead of any medication did not change the pattern of findings (data not shown).

**Table 3:** Association of Attention-Deficit/Hyperactivity Disorder (ADHD) Polygenic Risk Score (PRS) in Referred Youth With Academic Achievement, Controlling for Age, Sex, Medication Use, and Ancestry Components

ADHD PRS Threshold	Word Reading (n = 393)				Numerical Operations (n = 398)			
	*b*	*R*^*2*^ (%)	*F*	Permuted *p*	*b*	*R*^*2*^ (%)	*F*	Permuted *p*
*p* < 1.0 × 10^−6^	1.90	1.58	6.64	.0129	.49	.11	0.45	.5021
*p* < 1.0 × 10^−5^	1.05	.47	1.96	.1791	.06	.00	0.01	.9368
*p* < 1.0 × 10^−4^	.27	.03	0.13	.7186	−.23	.02	0.10	.7512
*p* < .001	.64	.19	0.77	.3966	−.03	.00	0.00	.9706
*p* < .01	−1.01	.47	1.94	.1807	−1.04	.52	2.06	.1451
***p* < .05**	**−2.11**	**2.05**	**8.68**	**.0043**	−1.88	1.67	6.74	.0101
***p* < .1**	**−2.04**	**1.99**	**8.42**	**.0051**	−1.82	1.61	6.52	.0112
***p* < .3**	−1.96	1.77	7.48	.0084	**−2.03**	**1.94**	**7.87**	**.0061**
***p* < .5**	**−2.01**	**1.88**	**7.94**	**.0066**	**−2.11**	**2.12**	**8.60**	**.0042**
***p* < 1**	**−2.04**	**1.92**	**8.09**	**.0057**	**−2.20**	**2.27**	**9.25**	**.0030**

Note: Boldface type indicates statistically significant findings after correction. Bonferroni corrected critical value = .0083.

Regarding discriminant validity, first we analyzed associations between ADHD PRSs and traits that were not expected to be associated with ADHD. As shown in Table S7 (available online), no significant associations or pattern of trend-level findings emerged for somatic complaints or social cognition. We then examined SCZ PRSs (Table S8, available online) and ASD PRSs (Table S9, available online) in relation to ADHD status and ADHD symptoms. No significant associations were found for either SCZ PRSs or ASD PRSs.

### Question 2. Association of ADHD PRSs With Phenotypes Beyond ADHD

ADHD PRSs were also associated with aggression and WM across diagnoses (Table 4). For aggression, significant associations were found at the three most stringent *p* value thresholds (*R*^*2*^ values, 1.90%–2.59%). For WM, three significant associations were found (*R*^*2*^ values, 2.26%–2.47%), though at the three most inclusive thresholds. Significant associations remained for aggression at two of the three significant thresholds after controlling for ADHD status, with slightly less explained variance (*R*^*2*^ values, 1.36%–1.99%). Associations with WM were unaffected (Table S6, available online). As with academic achievement, controlling for stimulants instead of all medications did not change results.

**Table 4:** Association of Attention-Deficit/Hyperactivity Disorder (ADHD) Polygenic Risk Score (PRS) in Referred Youth With Traits Beyond ADHD, Controlling for Age, Sex, and Ancestry Components

ADHD PRS Threshold	Aggressive Behavior (n = 394)				Working Memory Index^a^ (n = 394)			
	*b*	*R*^*2*^ (%)	*F*	Permuted *p*	*b*	*R*^*2*^ (%)	*F*	Permuted *p*
*p* < 1.0 × 10^−6^	**1.35**	**1.90**	**7.66**	**.0082**	.94	.42	1.69	.1982
*p* < 1.0 × 10^−5^	**1.58**	**2.59**	**10.52**	**.0019**	.11	.01	0.02	.8889
*p* < 1.0 × 10^−4^	**1.49**	**2.40**	**9.70**	**.0020**	−.09	.00	0.02	.9003
*p* < .001	.94	.99	3.97	.0471	−.29	.04	0.18	.6737
*p* < .01	.83	.77	3.05	.0823	−1.01	.54	2.18	.1468
*p* < .05	.31	.11	0.42	.5145	−1.69	1.47	5.97	.0162
*p* < .1	.41	.19	0.77	.3760	−1.76	1.64	6.66	.0113
*p* < .3	.44	.21	0.84	.3573	**−2.17**	**2.47**	**10.10**	**.0016**
*p* < .5	.23	.06	0.24	.6226	**−2.08**	**2.26**	**9.23**	**.0030**
*p* < 1	.19	.04	0.17	.6847	**−2.10**	**2.33**	**9.53**	**.0025**

Note: Boldface type indicates statistically significant findings after correction.

a Also controlled for medication use; Bonferroni corrected critical value = .0083.

### Biobank Replication

As shown in Table S10 (available online), ADHD PRSs were associated with ADHD diagnoses in adults. At the six most inclusive thresholds, a 1 SD increase in ADHD PRSs resulted in a 1.2-fold increase in the odds of having ADHD versus no ADHD. In a secondary analysis (also Table S10, available online), we found significant ORs of similar magnitude at the four most inclusive thresholds when patients with non-ADHD neuropsychiatric diagnoses were the reference group, further supporting ADHD PRS convergent validity in patients.

ADHD PRSs were also associated with educational attainment at all but one discovery sample threshold, with greater ADHD risk reducing the likelihood of college completion (ORs, 1.13–1.23) (Table S11, available online). Finally, ADHD PRSs were associated with an increased risk for SUDs at all discovery sample thresholds, with modest ORs (1.10–1.18) (Table S12, available online). Controlling for ADHD status did not change these two results in a meaningful way (Table S13, available online).

### Question 3. Multivariate Clinical Profile in Youths With a High ADHD Polygenic Burden

Based on the three most stringent discovery thresholds, youth with a high ADHD polygenic burden, on average, manifested a distinctive clinical profile compared with youths with a medium or low burden (Table S14, available online). Significant results were driven by a main effect of risk group; youths in the high polygenic burden group had a more severe multivariate pattern of psychopathology symptoms compared with youths in the low-risk group (*β* values = .21–.24, *p* ≤ .014). No significant differences were found between the medium-risk and low-risk groups.

**Figure 1:**
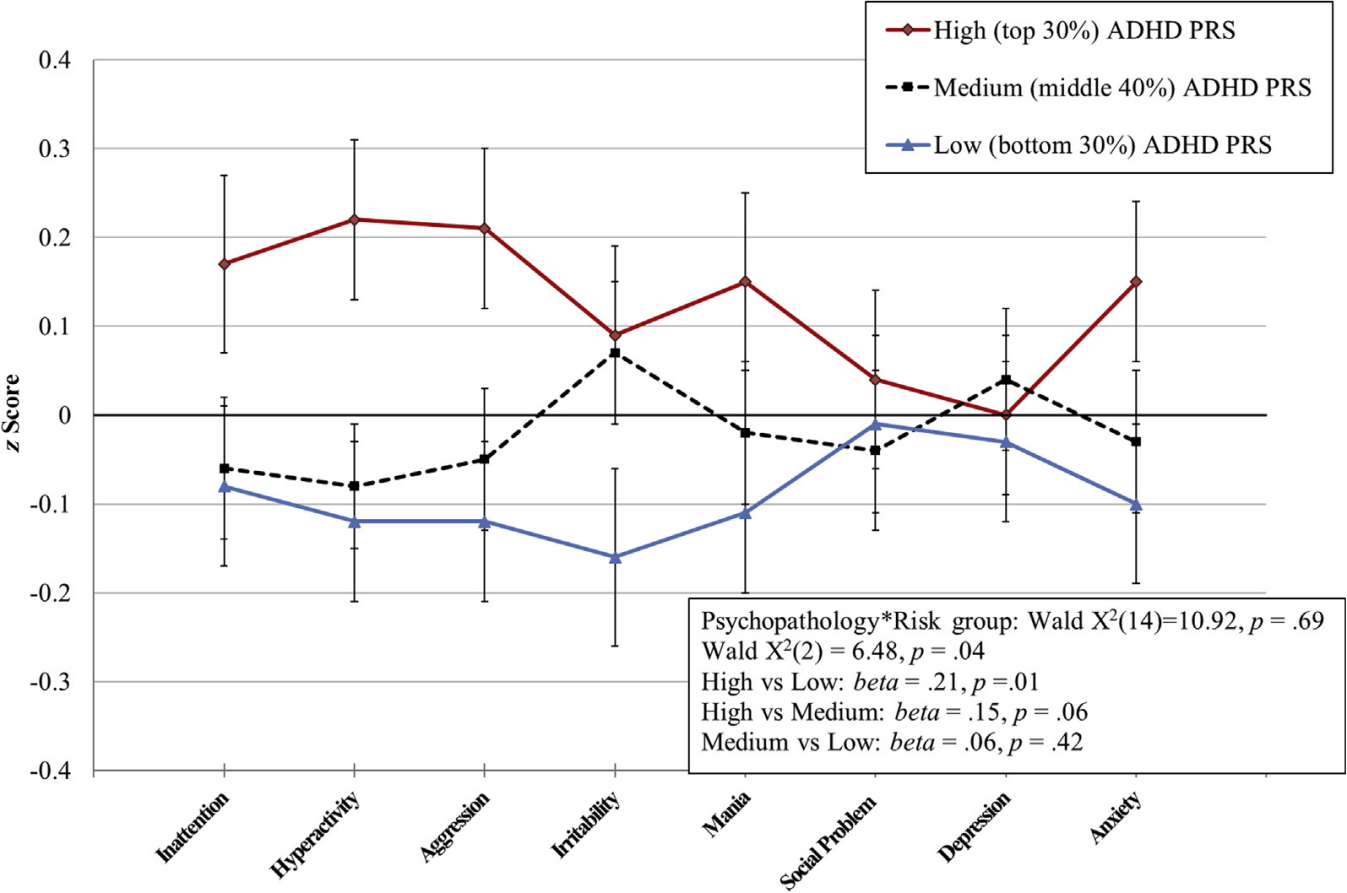
High and Low Levels of Attention-Deficit/Hyperactivity Disorder (ADHD) Polygenic Risk Are Characterized by Different Psychopathology Symptom Profiles in Referred Youth ***Note:****Mixed-effects modeling (based on discovery sample threshold* p *< 1.0 × 10*^*−4*^*). Results demonstrate a main effect for risk group, reflecting a significantly more severe psychopathology profile in youth with high ADHD polygenic risk compared with low-risk group. PRS = polygenic risk score. Please note color figures are available online.*

Figure 1 illustrates the results at the most inclusive significant threshold (*p* < 1.0 × 10^−4^). Although analyses did not yield a significant interaction between domain and risk group, this question may benefit from a larger sample with greater power to detect interactions. Post hoc contrasts suggested that youths with a high ADHD polygenic burden showed impairment on hyperactivity/impulsivity and aggression symptoms rather than all domains (hyperactivity: high versus low risk *b* = .34, *t* = 2.74, *p* = .01, high versus medium *b* = .29, *t* = 2.50, *p* = .01; aggression: high versus low risk *b* = .33, *t* = 2.57, *p* = .01, high versus medium *b* = .26, *t* = 2.14, *p* = .03).

## Discussion

This study provides what, to our knowledge, is the first translation of ADHD PRSs to a generalizable child and adolescent outpatient cohort. Results support the convergent and discriminant validity of ADHD PRSs with ADHD-related clinical phenotypes among youths referred for neuropsychiatric evaluation. Conceptual replication in adults confirmed a relationship with clinical ADHD diagnoses even versus other neuropsychiatric conditions. In youths and adults, ADHD PRSs were also associated with phenotypes beyond the *DSM*-defined ADHD construct (including WM and aggression in youths and SUDs in adults) that share its liability and have implications for outcome. Finally, youth with a high ADHD polygenic burden manifested a more severe clinical profile compared with youths with a lower burden. These findings justify and inform further studies aiming to leverage ADHD PRSs in child psychiatric practice.

ADHD PRSs in these analyses reflect the common genetic variation that collectively distinguished ADHD cases from non-ADHD controls in a GWAS of approximately 55,000 individuals.^5^ Although the biology underlying these small perturbations in molecular processes is not yet resolved, PRSs are unique among potential information sources in their ability to index this heritable liability. Our results demonstrate that variation in these risk scores tracks with the ADHD construct, broadly conceived, among youth outpatients with a range of psychopathology. Increasing ADHD polygenic burden was associated with increased odds of being diagnosed with full or subthreshold ADHD versus not receiving these diagnoses. Across patients, variation in ADHD PRSs also related to variation in ADHD symptoms and in academic impairment. Moreover, associations in outpatient youth were not indiscriminate. We found no relationship between ADHD PRSs and two traits not expected to be associated with ADHD (somatization and social cognition). A degree of specificity was also documented through lack of associations between SCZ and ASD PRSs with core ADHD phenotypes. Nonetheless, relationships emerged with cognitive (WM) and behavioral (aggression) phenotypes that lie outside the diagnostic boundaries of ADHD but that have been tied to ADHD liability in prior studies.^6^, ^40^

Analyses in adults conceptually replicated these findings. Our adult sample derived from the same catchment area as our youth cohort and similarly examined real-world clinical diagnoses, which are relevant to clinical translation. In adult patients, ADHD PRSs were associated with having an ADHD diagnosis, even versus having another neuropsychiatric condition. Results in adults also substantiated the relationship between ADHD PRSs and educational impairment, extending implications beyond grade school achievement to educational attainment in adulthood. Finally, in adults, ADHD PRSs were associated with SUD, which typically has onset later than the age of our youth sample and was not present in that cohort but which has a putative relationship to ADHD risk.^36^

For genomic information to have clinical utility as an objective risk indicator, precise tracking with *DSM* diagnoses is not necessary. Eventually, risk scores are expected to be refined to reflect more specific biological processes with implications for treatment, and these may link to a range of potential outcomes.^41^ Nonetheless, PRSs from *DSM*-based GWASs represent a useful anchor, as major categories of illness will likely contribute to the organizing principles of this evolving framework, given some specificity in the genomics and psychopharmacologic literatures.^41^ While the precision of PRS will increase with larger GWAS samples, our data raise the possibility that even the small-magnitude relationships found in our cohort could contribute to earlier and more efficient diagnosis and/or prompt consideration of treatments with benefits for ADHD. This possibility requires direct evaluation of PRSs in clinical trials as well as further clinical science. Regarding the latter, how exactly genomic risk scores should be calibrated must be determined and may benefit from examples in other medical fields (eg, see Khera *et al.*^42^). Capitalizing on ADHD PRSs as objective indicators will also require mapping phenotypic associations beyond those examined in this article, including traits relevant to mood disorders that partially share genetic risk with ADHD.^10^ Furthermore, the possibility that PRSs based on different GWAS thresholds (ie, different subgroups of ADHD-related variants) may relate to particular phenotypes should be considered, given that aggression and cognitive/academic skills in our sample were associated with PRS at different discovery sample cutoffs.

Finally, our data extend the empirical justification for evaluating ADHD PRSs as tools for clinical risk stratification. In the literature, impaired executive cognition has been associated with academic difficulties^14^, ^15^ and reduced occupational attainment^15^ over and above ADHD. Additionally, co-occurring aggression can create long-term psychosocial disruption.^16^, ^17^ In our youth cohort, variation in ADHD PRSs was associated with variation in these traits, highlighting possible means by which high ADHD PRSs could relate to particular patient outcomes. We also examined the implications of a high ADHD PRS directly in relation to psychopathology symptom profiles. For several discovery sample thresholds, referred youth with high ADHD polygenic burden manifested a more severe clinical profile. A nonsignificant interaction term prevented us from concluding that there was a relationship to particular symptom domains rather than generally increased severity; however, post hoc tests suggested that youths in the high-risk group had increased hyperactivity/impulsivity and aggression. Thus, the possibility that high ADHD PRSs relate to a particular symptom profile should be examined in larger samples. Regardless, the fact that being in the highest ADHD PRS strata was associated with clinical severity, even among referred youth, extends the rationale for considering PRS as a tool for risk stratification in the clinical setting.

To confirm the potential value of genomic data for this purpose, studies will need to document the longitudinal outcomes of referred youth with high ADHD polygenic burden and clarify the age of penetrance and potential mediators of associated phenotypes. For example, though ADHD onsets primarily in youth, prevention and intervention strategies will depend on whether being at high risk is associated with early and severe ADHD symptoms versus their gradual unfolding. Moreover, the fact that the majority of associations to phenotypes beyond ADHD remained after controlling for the diagnosis suggests that simply treating ADHD will not be sufficient for improving outcomes in high-risk youth. Rather, genetically informed prevention/early intervention programs that address specific outcomes should be considered, and such efforts must be appropriately timed. For instance, cost-effective targeting of high-risk youth for educational supports and substance prevention programs could be beneficial, but longitudinal confirmation of our data would suggest that increased academic support is needed during an earlier developmental window.

Limitations of our study should be acknowledged. First, the trade-off for collecting a large clinical cohort in a cost-efficient manner was a lack of structured diagnostic interviews. Yet, several factors support the integrity of our diagnoses. Our youth clinic is a training site at a teaching hospital where attention is given to differential diagnosis. Furthermore, κ values from blinded ratings showed high interclinician agreement, and we validated the ADHD diagnosis, once made, with structured interviews in a subset of patients. Furthermore, clinical diagnoses, though imprecise, are the criterion most relevant to clinical translation,^43^ and analyses in adults conceptually replicated key associations. Effect sizes for diagnoses in adults were slightly lower than in youth; this was likely due to the wider variety of clinicians and clinics from which they came and/or the fact that ADHD in youths and adults shares some, but not all, genetic underpinnings (see Faraone and Larsson^21^). Despite these factors, the convergence of electronic health record diagnostic codes with gold standard clinician ratings has been established in this biobank previously for ADHD^44^ and other conditions,^45^ and both adults and youth with ADHD were included in the source GWAS.^5^ Thus, the significant results in our adult sample represent an appropriate corroboration of primary findings.

Second, we cannot rule out the possibility that medication masked some variation in symptoms and test performance, even after adjusting for covariates. Whereas a medication-naïve sample would have been preferable, we did not limit enrollment to preserve the generalizability of findings. Similarly, we note that slightly more than half of the youth in our sample had previously received treatment via psychotherapy or medication. A source clinic such as ours, which provides comprehensive neuropsychiatric evaluations for purposes of differential diagnosis and school and treatment planning, represents a setting in which genomic data would be highly relevant. Thus, we included all consecutive referrals to the clinic, regardless of treatment history. Third, we used ASD PRS to address specificity/discriminant validity because it related to a childhood-onset condition for which most of our youth have passed through the age of risk. Nonetheless, the discovery GWAS for ASD is small, and associations between ASD PRS and ADHD in our sample should be revisited when the size of that GWAS increases. Fourth, as discussed, we may have lacked statistical power to detect interactions in our mixed effects models and thus cannot speak to whether a high polygenic burden is characterized by greater symptoms in specific psychopathology domains versus greater symptoms more generally. Finally, we note that the current analyses were limited to participants of European ancestries. Translation to more racially and ethnically representative samples is critical and will benefit from both statistical advances (eg, see Seldin *et al.*^46^) and collection of samples from other populations (eg, see Dalvie *et al.*^47^).

Despite these issues, our results provide novel evidence that ADHD PRSs are relevant to phenotypes that reflect the broad ADHD construct among child psychiatric outpatients. Additionally, a relatively high PRS burden in outpatients is associated with greater clinical severity. It has long been hoped that advances in genetics would have a positive impact on child psychiatry,^48^ and data from recent GWASs are creating opportunities that have not previously existed. The current findings help to advance the foundational clinical science needed to translate ADHD PRSs to clinical practice.
