# Supplementary material for: Translating Discoveries in Attention-Deficit/Hyperactivity Disorder Genomics to an Outpatient Child and Adolescent Psychiatric Cohort
Source: J Am Acad Child Adolesc Psychiatry. 2020 Aug;59(8):964–77. doi: 10.1016/j.jaac.2019.08.004 (PMC7408479; doi:10.1016/j.jaac.2019.08.004)
Supplement: Supplementary Material [file mmc1.docx]

**Supplement 1**

Clinical diagnoses and their reliability:

Our source clinic is an accredited clinical psychology training site, with an emphasis on thorough and accurate diagnosis. Clinical diagnoses are made based on interviews with parents/guardians and patients, review of medical records, and rating scales. They were coded in the primary record if full DSM-IV-TR criteria were met, with two exceptions. First, an ADHD diagnosis was allowed to co-occur with autism spectrum disorder (ASD), in anticipation of DSM-5. Second, oppositional defiant and conduct disorders (ODD/CD) were not coded in the primary record unless they were the sole diagnosis. For research purposes, diagnoses of ODD/CD were coded in line with DSM-IV criteria, by supplementing the record with diagnoses based on symptom-level reports from the extended record.

We examined the reliability of major diagnostic groupings in separate reliability studies for ADHD, ASD, mood disorders and psychosis-related diagnoses. For each group, a licensed clinical psychologist blindly rated diagnoses from 30 youth, half of whom were randomly selected from among referrals with the diagnosis and half from those without. Results showed good inter-rater reliability between the rater and the clinical diagnosis (Cohen’s Kappa =.93 for ADHD, ASD, and mood disorders; 95% CI: [.80-1.06] and .80; 95% CI: [.59-1.01] for a psychosis-related diagnosis). Additionally, to confirm validity of the ADHD diagnosis once made, we examined the convergence of diagnoses of ADHD from our clinic and diagnoses made from a semi-structured diagnostic interview (i.e. KSADS-E) in twelve youth through a separate research project. Clinician ADHD diagnoses were confirmed by the interview in 100% of these cases.

QC of genomic data and calculation of PRS in youth cohort:

Based on standard filters and QC procedures, we excluded SNPs with the following characteristics: excessive missingness, non-polymorphic, Hardy-Weinberg disequilibrium test *p*<10^-6^, non-autosomal, or minor allele frequency (MAF)<0.01. Subsequently, 283,788 genotyped variants were retained. Samples were excluded due to sex mismatch or relatedness (sibling), with the first sibling evaluated retained for analysis. Markers were imputed using Minimac3 using data from the 1000 Genomes project (phase 3) as the haplotype reference panel. Imputed best guess genotypes were filtered to retain only high quality common markers (INFO>0.8 & MAF>0.05), yielding 2,489,398 variants. Principal component analysis (PCA) including individuals in the 1000 Genomes Project was used to exclude non-Europeans (n=183 probands). To calculate risk scores, discovery data were filtered for MAF>0.01 & INFO>0.8. SNPs were excluded if they were ambiguous/asymmetric, had duplicate position, or had inconsistent alleles in LOGIC. LD-clumping (--clump-kb 5000 --clump-r2 0.2) in PLINK was used to extract an independent set of SNPs for PRS calculation.

**Supplemental Tables**

**Table S1: In Referred Youth (n=433): Rates of Psychotropic Medication Use**

| **Category** | **N (%)** |
| --- | --- |
| Stimulants | 101 (23.3) |
| Non-Stimulants for ADHD  (e.g. atomoxetine) | 44 (10.2) |
| Atypical Antipsychotic | 41 (9.5) |
| SSRI | 72 (16.6) |
| Non-SSRI Antidepressant | 19 (4.4) |
| Benzodiazepine | 11 (2.5) |
| Other | 25 (5.8) |

Note: some youth were taking more than one type of medication; ADHD= Attention-Deficit/Hyperactivity Disorder; SSRI= Selective Serotonin Reuptake Inhibitor.

**Table S2: Number of SNPs at each *p*-value Threshold for ADHD, ASD and SCZ PRS**

| *p*T< | ADHD | ASD | SCZ |
| --- | --- | --- | --- |
| 1 | 84,528 | 49660 | 48090 |
| 0.5 | 60,375 | 35561 | 36450 |
| 0.3 | 43,303 | 25547 | 28055 |
| 0.1 | 20,113 | 11791 | 15203 |
| 0.05 | 12,273 | 7023 | 10410 |
| 0.01 | 3,937 | 2057 | 4509 |
| 0.001 | 831 | 381 | 1537 |
| 0.0001 | 203 | 63 | 656 |
| 0.00001 | 66 | 20 | 307 |
| 0.000001 | 23 | 5^a^ | 157 |
| 0.0000001 | 9^a^ | 1^a^ | 101 |
| 0.00000005 | 6^a^ | 1^a^ | 90 |

Note: ADHD= Attention-Deficit/Hyperactivity Disorder; ASD= Autism Spectrum Disorder; PRS= Polygenic Risk Scores; SCZ= Schizophrenia; SNP= Single Nucleotide Polymorphism; ^a^ Scores not analyzed due to limited numbers of SNPs.

| **Table S3. In Referred Youth: Correlations between ADHD PRS Derived from Increasingly Inclusive Discovery Sample *p*-value Thresholds** | | | | | | | | | | |
| --- | --- | --- | --- | --- | --- | --- | --- | --- | --- | --- |
|  | **ADHD PRS at each *p*-value threshold (n=433)** | | | | | | | | | |
| **ADHD PRS Threshold** | **1.0 x 10^-6^** | **1.0 x 10^-5^** | **1.0 x 10^-4^** | **0.001** | **0.01** | **0.05** | **0.1** | **0.3** | **0.5** | **1.0** |
| **1.0 x 10^-6^** | 1.00 | - | - | - | - | - | - | - | - | - |
| **1.0 x 10^-5^** | .70 | 1.00 | - | - | - | - | - | - | - | - |
| **1.0 x 10^-4^** | .44 | .64 | 1.00 | - | - | - | - | - | - | - |
| **0.001** | .26 | .44 | .64 | 1.00 | - | - | - | - | - | - |
| **0.01** | .20 | .31 | .49 | .67 | 1.00 | - | - | - | - | - |
| **0.05** | .14 | .22 | .36 | .51 | .76 | 1.00 | - | - | - | - |
| **0.1** | .10 | .20 | .32 | .47 | .68 | .91 | 1.00 | - | - | - |
| **0.3** | .07 | .15 | .24 | .41 | .59 | .82 | .90 | 1.00 | - | - |
| **0.5** | .06 | .14 | .24 | .39 | .58 | .81 | .88 | .98 | 1.00 | - |
| **1.0** | .07 | .15 | .24 | .39 | .58 | .80 | .87 | .97 | .99 | 1.00 |

Note: ADHD= Attention/Deficit-Hyperactivity Disorder; PRS = Polygenic Risk Scores.

| **Table S4. In Referred Youth: Multinomial Logistic Regression Examining the Association between ADHD PRS and ADHD Status (definite, sub-threshold and no ADHD)** | | | | | | | |
| --- | --- | --- | --- | --- | --- | --- | --- |
|  | **Definite ADHD (n=255) vs sub-threshold ADHD (n=68)** | | **Definite ADHD (n=255) vs no ADHD (n=110)** | | **Overall test statistic** | | |
| **ADHD PRS Threshold** | **RRR** | **Permuted *p*-value** | **RRR** | **Permuted *p*-value** | **Pseudo R^2^(%)** | **Wald X^2^(2)** | ***p*-value** |
| ***p* < 1.0 x 10^-6^** | 1.33 | .0491 | 1.49 | .0011 | 1.51 | 11.88 | .0026 |
| ***p* < 1.0 x 10^-5^** | 1.14 | .3760 | 1.41 | .0054 | 1.00 | 7.95 | .0187 |
| ***p* < 1.0  x 10^-4^** | 1.13 | .3851 | 1.29 | .0314 | 0.59 | 4.73 | .0938 |
| ***p* < 0.001** | 0.93 | .5778 | 1.24 | .0664 | 0.54 | 4.33 | .1147 |
| ***p* < 0.01** | 0.96 | .7499 | 1.42 | .0026 | 1.21 | 9.49 | .0087 |
| ***p* < 0.05** | 0.85 | .2439 | 1.27 | .0431 | 0.85 | 6.82 | .0331 |
| ***p* < 0.1** | 0.90 | .4573 | 1.32 | .0177 | 0.94 | 7.47 | .0239 |
| ***p* < 0.3** | 0.93 | .5983 | 1.31 | .0211 | 0.82 | 6.60 | .0369 |
| ***p* < 0.5** | 0.93 | .6081 | 1.37 | .0078 | 1.06 | 8.43 | .0148 |
| ***p* < 1** | 0.94 | .6382 | 1.40 | .0051 | 1.17 | 9.27 | .0097 |
| Note: ADHD= Attention/Deficit-Hyperactivity Disorder; PRS = Polygenic Risk Score; RRR = Relative Risk Ratio. | | | | | | | |

| **Table S5. In Referred Youth: Post-Hoc Association of ADHD PRS with Inattention and Hyperactivity/Impulsivity Symptoms, Controlling for Age, Sex and Ancestry** | | | | | | | | | | | | | |
| --- | --- | --- | --- | --- | --- | --- | --- | --- | --- | --- | --- | --- | --- |
|  | **Primary analyses** | | | | | **Secondary analyses** | | | | | | | |
|  | | **ADH Problems scale**  **(n=394)** | | | | **Inattention Symptoms**  **(n=385)** | | | | **Hyperactivity/Impulsivity symptoms**  **(n=386)** | | | |
| **ADHD PRS Threshold** | | ***b*** | **R^2^(%)** | **F-value** | **Permuted *p*-value** | ***b*** | **R^2^(%)** | **F-value** | **Permuted *p*-value** | ***b*** | **R^2^(%)** | **F-value** | **Permuted *p*-value** |
| ***p* < 1.0 x 10^-6^** | | 1.46 | 2.93 | 11.83 | .0007 | 0.78 | 1.28 | 4.94 | .0259 | 0.84 | 1.44 | 6.32 | .0188 |
| ***p* < 1.0 x 10^-5^** | | 1.28 | 2.24 | 8.97 | .0027 | 0.66 | 0.94 | 3.63 | .0540 | 0.90 | 1.74 | 7.65 | .0114 |
| ***p* < 1.0  x 10^-4^** | | 1.13 | 1.84 | 7.34 | .0069 | 0.68 | 1.01 | 3.91 | .0452 | 0.97 | 2.00 | 8.81 | .0063 |
| ***p* < 0.001** | | 0.62 | 0.57 | 2.23 | .1424 | 0.26 | 0.15 | 0.59 | .4397 | 0.59 | 0.76 | 3.31 | .0856 |
| ***p* < 0.01** | | 0.46 | 0.32 | 1.25 | .2620 | 0.27 | 0.16 | 0.61 | .4290 | 0.54 | 0.62 | 2.69 | .1201 |
| ***p* < 0.05** | | 0.02 | 0.00 | 0.00 | .9556 | 0.11 | 0.01 | 0.10 | .7472 | 0.35 | 0.27 | 1.18 | .3106 |
| ***p* < 0.1** | | 0.07 | 0.01 | 0.03 | .8561 | 0.30 | 0.09 | 0.34 | .5575 | 0.26 | 0.15 | 0.65 | .4518 |
| ***p* < 0.3** | | 0.34 | 0.17 | 0.66 | .4186 | 0.47 | 0.51 | 1.95 | .1581 | 0.44 | 0.44 | 1.93 | .1989 |
| ***p* < 0.5** | | 0.26 | 0.10 | 0.40 | .5279 | 0.48 | 0.54 | 2.08 | .1447 | 0.47 | 0.51 | 2.22 | .1657 |
| ***p* < 1** | | 0.24 | 0.09 | 0.34 | .5609 | 0.51 | 0.60 | 2.30 | .1265 | 0.47 | 0.50 | 2.17 | .1686 |
| Note: Shaded rows indicate statistically significant findings after correction. ADH = Attention Deficit/Hyperactivity; ADHD= Attention/Deficit-Hyperactivity Disorder; PRS= Polygenic Risk Scores; Primary analyses: Bonferroni corrected critical value = .0083; Secondary analyses: Bonferroni corrected critical value = .0250. | | | | | | | | | | | | | |

| **Table S6. Sensitivity Analysis in Referred Youth: Re-Analysis of Significant Associations After Controlling for ADHD Status** | | | | | |
| --- | --- | --- | --- | --- | --- |
| **Domain** | **ADHD PRS Threshold** | ***b*** | **R²(%)** | **F-value** | **Permuted *p*-value** |
| **Aggressive behavior**  **(n=394)** | ***p* < 1.0 x 10^-6^** | 1.15 | 1.36 | 5.58 | .0234 |
|  | ***p* < 1.0  x 10^-5^** | 1.40 | 1.99 | 8.22 | .0050 |
|  | ***p* < 1.0 x 10^-4^** | 1.34 | 1.92 | 7.91 | .0054 |
|  |  |  |  |  |  |
| **Working Memory Index**  **(n=394)** | ***p* < 0.3** | -2.16 | 2.38 | 9.74 | .0025 |
|  | ***p* < 0.5** | -2.06 | 2.17 | 8.86 | .0035 |
|  | ***p* < 1** | -2.09 | 2.24 | 9.15 | .0032 |
|  |  |  |  |  |  |
| **Word Reading**  **(n=393)** | ***p* < 0.05** | -2.16 | 2.12 | 8.94 | .0041 |
|  | ***p* < 0.1** | -2.10 | 2.07 | 8.73 | .0040 |
|  | ***p* < 0.5** | -2.08 | 1.96 | 8.25 | .0054 |
|  | ***p* < 1** | -2.12 | 2.00 | 8.44 | .0047 |
|  |  |  |  |  |  |
| **Numerical Operations**  **(n=398)** | ***p* < 0.3** | -1.85 | 1.59 | 6.50 | .0117 |
|  | ***p* < 0.5** | -1.93 | 1.72 | 7.02 | .0092 |
|  | ***p* < 1** | -2.00 | 1.84 | 7.52 | .0067 |

Note: Shaded rows indicate statistically significant findings after correction; ADHD= Attention/Deficit-Hyperactivity Disorder; PRS= Polygenic Risk Scores.

| **Table S7. In Referred Youth: Association of ADHD PRS with Traits to Establish Divergent Validity (Somatic Complaints and Social Cognition), Controlling for Age, Sex, and Ancestry** | | | | | | | | |
| --- | --- | --- | --- | --- | --- | --- | --- | --- |
|  | **Somatic Complaints**  **(n=394)** | | | | **Social Cognition**  **(n= 338)** | | | |
| **ADHD PRS Threshold** | ***b*** | **R^2^(%)** | **F-value** | **Permuted *p*-value** | ***b*** | **R^2^(%)** | **F-value** | **Permuted *p*-value** |
| ***p* < 1.0 x 10^-6^** | 0.12 | 0.02 | 0.08 | .7883 | 0.56 | 0.70 | 2.36 | .1214 |
| ***p* < 1.0 x 10^-5^** | 0.15 | 0.03 | 0.11 | .7458 | 0.24 | 0.13 | 0.43 | .5057 |
| ***p* < 1.0  x 10^-4^** | 0.50 | 0.33 | 1.35 | .2568 | 0.14 | 0.05 | 0.15 | .6945 |
| ***p* < 0.001** | 0.03 | 0.00 | 0.01 | .9410 | -0.08 | 0.01 | 0.05 | .8291 |
| ***p* < 0.01** | 0.52 | 0.37 | 1.50 | .2265 | -0.35 | 0.27 | 0.91 | .3385 |
| ***p* < 0.05** | -0.32 | 0.15 | 0.59 | .4509 | -0.45 | 0.47 | 1.58 | .2117 |
| ***p* < 0.1** | -0.17 | 0.04 | 0.16 | .6899 | -0.38 | 0.34 | 1.16 | .2826 |
| ***p* < 0.3** | -0.04 | 0.00 | 0.01 | .9275 | -0.04 | 0.00 | 0.01 | .9023 |
| ***p* < 0.5** | -0.15 | 0.03 | 0.13 | .7256 | -0.11 | 0.03 | 0.10 | .7545 |
| ***p* < 1** | -0.14 | 0.03 | 0.11 | .7398 | -0.14 | 0.05 | 0.17 | .6808 |
| Note: ADHD= Attention/Deficit-Hyperactivity Disorder; PRS= Polygenic Risk Scores; Critical value = .05. | | | | | | | | |

| **Table S8. In Referred Youth: Prediction of ADHD Diagnosis and ADHD Symptoms by the SCZ PRS Using Logistic and Linear Regression, Controlling for Age, Sex, and Ancestry** | | | | | | | | | | |
| --- | --- | --- | --- | --- | --- | --- | --- | --- | --- | --- |
| **SCZ PRS Threshold** | **ADHD spectrum (n=323) vs. all others in clinical cohort (n=110)** | | | | | **ADH Problems**  **(n= 394)** | | | | |
|  | **OR** | **95% CI** | **Pseudo R^2^(%)** | **Wald X^2^(1)** | **Permuted**  ***p-*value** | ***b*** | **R^2^(%)** | **F-value** | | **Permuted *p*-value** |
| ***p* < 5.0 x 10^-8^** | 0.88 | 0.71 – 1.10 | 0.26 | 1.26 | .2670 | 0.00 | 0.00 | 0.00 | .9966 | |
| ***p* < 1.0 x 10^-7^** | 0.91 | 0.73 – 1.14 | 0.14 | 0.69 | .4108 | 0.14 | 0.03 | 0.11 | .7315 | |
| ***p* < 1.0 x 10^-6^** | 1.04 | 0.83 – 1.29 | 0.02 | 0.10 | .7566 | 0.46 | 0.31 | 1.20 | .2782 | |
| ***p* < 1.0 x 10^-5^** | 1.00 | 0.81 – 1.25 | 0.00 | 0.00 | .9774 | 0.25 | 0.09 | 0.36 | .5548 | |
| ***p* < 1.0 x 10^-4^** | 0.91 | 0.73 – 1.13 | 0.15 | 0.74 | .3926 | 0.49 | 0.34 | 1.33 | .2549 | |
| ***p* < 0.001** | 0.99 | 0.79 – 1.25 | 0.00 | 0.00 | .9529 | 0.45 | 0.28 | 1.08 | .2982 | |
| ***p* < 0.01** | 1.19 | 0.95 – 1.48 | 0.45 | 2.20 | .1419 | 0.73 | 0.73 | 2.90 | .0928 | |
| ***p* < 0.05** | 1.18 | 0.94 – 1.48 | 0.39 | 1.93 | .1681 | 0.53 | 0.37 | 1.47 | .2309 | |
| ***p* < 0.1** | 1.22 | 0.97 – 1.53 | 0.58 | 2.81 | .0934 | 0.64 | 0.56 | 2.19 | .1457 | |
| ***p* < 0.3** | 1.21 | 0.96 – 1.53 | 0.53 | 2.56 | .1083 | 0.76 | 0.77 | 3.04 | .0849 | |
| ***p* < 0.5** | 1.22 | 0.96 – 1.54 | 0.55 | 2.67 | .0985 | 0.77 | 0.78 | 3.08 | .0837 | |
| ***p* < 1** | 1.22 | 0.96 – 1.54 | 0.53 | 2.60 | .1037 | 0.81 | 0.83 | 3.28 | .0737 | |
| Note: ADH= Attention Deficit/Hyperactivity; ADHD= Attention/Deficit-Hyperactivity Disorder; CI= Confidence Interval; OR= Odds Ratio; PRS= Polygenic Risk Scores; SCZ= Schizophrenia; Critical value = .05. | | | | | | | | | | |

| **Table S9. In Referred Youth: Prediction of ADHD Diagnosis and ADHD Symptoms by the ASD PRS Using Logistic and Linear Regression, Controlling for Age, Sex, and Ancestry** | | | | | | | | | |
| --- | --- | --- | --- | --- | --- | --- | --- | --- | --- |
| **ASD PRS Threshold** | **Definite ADHD (n=323) vs. all others in clinical cohort (n=110)** | | | | | **ADH Problems**  **(n= 394)** | | | |
|  | **OR** | **95% CI** | **Pseudo R^2^(%)** | **Wald X^2^(1)** | **Permuted**  ***p-*value** | ***b*** | **R^2^(%)** | **F-value** | **Permuted *p*-value** |
| ***p* < 1.0 x 10^-5^** | 1.02 | 0.81 – 1.28 | 0.01 | 0.04 | .8465 | 0.21 | 0.06 | 0.23 | .6274 |
| ***p* < 1.0 x 10^-4^** | 1.00 | 0.80 – 1.25 | 0.00 | 0.00 | .9716 | 0.14 | 0.03 | 0.11 | .7333 |
| ***p* < 0.001** | 1.02 | 0.82 – 1.27 | 0.00 | 0.03 | .8707 | 0.24 | 0.08 | 0.31 | .5810 |
| ***p* < 0.01** | 1.16 | 0.93 – 1.44 | 0.34 | 1.66 | .2016 | 0.37 | 0.19 | 0.75 | .3888 |
| ***p* < 0.05** | 1.03 | 0.83 – 1.29 | 0.01 | 0.08 | .7850 | -0.06 | 0.01 | 0.02 | .8833 |
| ***p* < 0.1** | 1.05 | 0.84 – 1.31 | 0.04 | 0.19 | .6661 | -0.06 | 0.01 | 0.02 | .8830 |
| ***p* < 0.3** | 1.07 | 0.86 – 1.35 | 0.08 | 0.38 | .5447 | 0.08 | 0.01 | 0.03 | .8536 |
| ***p* < 0.5** | 1.07 | 0.86 – 1.35 | 0.08 | 0.38 | .5485 | -0.05 | 0.00 | 0.01 | .9051 |
| ***p* < 1** | 1.06 | 0.85 – 1.33 | 0.05 | 0.26 | .6224 | -0.03 | 0.00 | 0.01 | .9376 |
| Note: ADH= Attention Deficit/Hyperactivity; ADHD= Attention/Deficit-Hyperactivity Disorder; ASD= Autism Spectrum Disorder; CI= Confidence Interval; OR= Odds Ratio; PRS= Polygenic Risk Scores; Critical value = .05. | | | | | | | | | |

| **Table S10. In Adult Biobank Sample: Association of ADHD PRS with ADHD Diagnoses, Controlling for Age, Sex, Ancestry and Genotyping Wave** | | | | | | | | | | |
| --- | --- | --- | --- | --- | --- | --- | --- | --- | --- | --- |
|  | **Primary analyses:**  **ADHD (n = 273) vs.**  **all other Biobank patients (n = 4867)** | | | | | **Secondary analyses:**  **ADHD (n = 273) vs.**  **Patients w/ other neuropsychiatric diagnoses (n = 2661)** | | | | |
| **Discovery Sample Threshold** | **Odds ratio** | **95% CI** | **Pseudo R^2^(%)** | **Wald X^2^** | **Permuted *p*-value** | **Odds ratio** | **95% CI** | **Pseudo R^2^(%)** | **Wald X^2^** | **Permuted *p*-value** |
| ***p* < 1.0 x 10^-6^** | 1.00 | 0.88 – 1.13 | 0.00 | 0.00 | .9525 | 0.95 | 0.84 – 1.08 | 0.03 | 0.58 | .4437 |
| ***p* < 1.0 x 10^-5^** | 1.00 | 0.88 – 1.13 | 0.00 | 0.00 | .9649 | 0.97 | 0.85 – 1.10 | 0.02 | 0.30 | .5882 |
| ***p* < 1.0  x 10^-4^** | 0.98 | 0.87 – 1.11 | 0.00 | 0.09 | .7597 | 0.94 | 0.83 – 1.07 | 0.04 | 0.78 | .3725 |
| ***p* < 0.001** | 1.07 | 0.95 – 1.21 | 0.06 | 1.24 | .2590 | 1.04 | 0.92 – 1.18 | 0.02 | 0.40 | .5199 |
| ***p* < 0.01** | 1.17 | 1.03 – 1.32 | 0.28 | 5.97 | .0121 | 1.13 | 1.00 – 1.29 | 0.20 | 3.58 | .0549 |
| ***p* < 0.05** | 1.17 | 1.04 - 133 | 0.30 | 6.38 | .0102 | 1.17 | 1.02 – 1.33 | 0.30 | 5.43 | .0206 |
| ***p* < 0.1** | 1.19 | 1.05 – 1.35 | 0.35 | 7.49 | .0058 | 1.20 | 1.05 – 1.37 | 0.40 | 7.27 | .0076 |
| ***p* < 0.3** | 1.19 | 1.05 – 1.34 | 0.34 | 7.25 | .0008 | 1.20 | 1.05 – 1.37 | 0.41 | 7.49 | .0061 |
| ***p* < 0.5** | 1.21 | 1.07 – 1.37 | 0.42 | 9.04 | .0028 | 1.22 | 1.07 – 1.39 | 0.50 | 9.11 | .0018 |
| ***p* < 1** | 1.20 | 1.06 – 1.36 | 0.38 | 8.05 | .0044 | 1.21 | 1.06 – 1.38 | 0.44 | 8.06 | .0044 |
| Note: Shaded rows indicate statistically significant findings after correction. ADHD= Attention/Deficit-Hyperactivity Disorder; CI= Confidence Interval; OR= Odds Ratio; PRS= Polygenic Risk Scores; Bonferroni corrected critical value = .0167. | | | | | | | | | | |

| **Table S11.  In Adult Biobank Sample (Ages 24-60): Association of ADHD PRS with College Completion, Controlling for Age, Sex, Ancestry and Genotyping Wave** | | | | | | |  |
| --- | --- | --- | --- | --- | --- | --- | --- |
|  | **No college (n = 688) vs. college (n = 1580)** | | | | |  | |
| **ADHD PRS Threshold** | **Odds ratio** | **95% CI** | **Pseudo R^2^(%)** | **Wald X^2^** | **Permuted*p*-value** | |  |
| ***p* < 1.0 x 10^-6^** | 1.14 | 1.04 – 1.26 | 0.28 | 7.81 | .0043 | |  |
| ***p* < 1.0 x 10^-5^** | 1.13 | 1.03 – 1.24 | 0.23 | 6.26 | .0105 | |  |
| ***p* < 1.0 x 10^-4^** | 1.08 | 0.99 – 1.19 | 0.11 | 2.86 | .0871 | |  |
| ***p* < 0.001** | 1.15 | 1.05 – 1.26 | 0.32 | 8.95 | .0027 | |  |
| ***p* < 0.01** | 1.15 | 1.05 – 1.27 | 0.32 | 8.81 | .0026 | |  |
| ***p* < 0.05** | 1.19 | 1.08 – 1.30 | 0.47 | 12.81 | .0005 | |  |
| ***p* < 0.1** | 1.23 | 1.12 – 1.35 | 0.72 | 19.58 | <.0001 | |  |
| ***p* < 0.3** | 1.21 | 1.10 – 1.33 | 0.59 | 16.23 | <.0001 | |  |
| ***p* < 0.5** | 1.22 | 1.11 – 1.34 | 0.63 | 17.24 | <.0001 | |  |
| ***p* < 1** | 1.22 | 1.11 – 1.34 | 0.61 | 16.61 | <.0001 | |  |
| Note: Shaded rows indicate statistically significant findings after correction. ADHD= Attention/Deficit-Hyperactivity Disorder; CI= Confidence Interval; OR= Odds Ratio; PRS= Polygenic Risk Scores; Bonferroni corrected critical value = .0167 | | | | | | |  |

| **Table S12. In Adult Biobank Sample: Association of ADHD PRS with SUD Controlling for Age, Sex, Ancestry and Genotyping Wave** | | | | | |  |
| --- | --- | --- | --- | --- | --- | --- |
|  | **SUD ( n =1089) vs. no SUD (n = 4051)** | | | | | |
| **ADHD PRS Threshold** | **Odds Ratio** | **95% CI** | **Pseudo R^2^(%)** | **Wald X^2^(1)** | **Permuted *p*-value** | |
| ***p* < 1.0 x 10^-6^** | 1.15 | 1.07 – 1.23 | 0.28 | 15.13 | .0002 | |
| ***p* < 1.0 x 10^-5^** | 1.15 | 1.07 – 1.23 | 0.28 | 15.95 | .0001 | |
| ***p* < 1.0 x 10^-4^** | 1.13 | 1.06 – 1.21 | 0.24 | 12.87 | .0004 | |
| ***p* < 0.001** | 1.18 | 1.10 – 1.26 | 0.40 | 21.19 | <.0001 | |
| ***p* < 0.01** | 1.14 | 1.06 – 1.22 | 0.26 | 14.00 | <.0001 | |
| ***p* < 0.05** | 1.10 | 1.02 – 1.17 | 0.13 | 7.10 | .0091 | |
| ***p* < 0.1** | 1.11 | 1.04 – 1.19 | 0.17 | 8.96 | .0020 | |
| ***p* < 0.3** | 1.11 | 1.03 – 1.18 | 0.16 | 8.33 | .0031 | |
| ***p* < 0.5** | 1.12 | 1.05 – 1.20 | 0.20 | 10.64 | .0004 | |
| ***p* < 1** | 1.12 | 1.04 – 1.20 | 0.19 | 10.33 | .0006 | |
| Note: Shaded rows indicate statistically significant findings after correction. ADHD= Attention/Deficit-Hyperactivity Disorder; CI= Confidence Interval; OR= Odds Ratio; PRS= Polygenic Risk Scores; SUD= Substance Use Disorder; Bonferroni corrected critical value = .0167. | | | | | | |

| **Table S13. Sensitivity Analysis in Adult Biobank: Re-Analysis of Significant Associations After Controlling for ADHD Status** | | | | | | |
| --- | --- | --- | --- | --- | --- | --- |
| **Outcome** | **ADHD PRS Threshold** | **Odds Ratio** | **95% CI** | **Pseudo R^2^(%)** | **Wald X^2^(1)** | **Permuted *p*-value** |
| **SUD**  **(n =1089)**  **vs. no SUD**  **(n = 4051)** | ***p* < 1.0 x 10^-6^** | 1.15 | 1.07 – 1.23 | 0.30 | 15.56 | <.0001 |
|  | ***p* < 1.0 x 10^-5^** | 1.15 | 1.08 – 1.23 | 0.31 | 16.41 | .0001 |
|  | ***p* < 1.0 x 10^-4^** | 1.14 | 1.06 – 1.22 | 0.25 | 13.32 | .0003 |
|  | ***p* < 0.001** | 1.17 | 1.10 – 1.26 | 0.39 | 20.68 | <.0001 |
|  | ***p* < 0.01** | 1.13 | 1.06 – 1.21 | 0.24 | 12.48 | .0003 |
|  | ***p* < 0.05** | 1.09 | 1.02 – 1.17 | 0.11 | 5.93 | .0152 |
|  | ***p* < 0.1** | 1.10 | 1.03 – 1.18 | 0.14 | 7.52 | .0058 |
|  | ***p* < 0.3** | 1.10 | 1.02 – 1.18 | 0.13 | 6.98 | .0076 |
|  | ***p* < 0.5** | 1.11 | 1.04 – 1.19 | 0.17 | 8.91 | .0013 |
|  | ***p* < 1** | 1.11 | 1.04 – 1.19 | 0.17 | 8.74 | .0017 |
| **No college**  **(n = 688)**  **vs.**  **college**  **(n = 1580)** | ***p* < 1.0 x 10^-6^** | 1.15 | 1.04 – 1.26 | 0.28 | 7.97 | .0040 |
|  | ***p* < 1.0 x 10^-5^** | 1.13 | 1.03 – 1.24 | 0.22 | 6.32 | .0101 |
|  | ***p* < 0.001** | 1.15 | 1.04 – 1.26 | 0.19 | 8.10 | .0035 |
|  | ***p* < 0.01** | 1.15 | 1.04 – 1.26 | 0.19 | 8.23 | .0040 |
|  | ***p* < 0.05** | 1.18 | 1.08 – 1.30 | 0.45 | 12.42 | .0005 |
|  | ***p* < 0.1** | 1.23 | 1.12 – 1.35 | 0.68 | 18.85 | <.0001 |
|  | ***p* < 0.3** | 1.21 | 1.10 – 1.33 | 0.55 | 15.34 | <.0001 |
|  | ***p* < 0.5** | 1.21 | 1.10 – 1.33 | 0.59 | 16.25 | <.0001 |
|  | ***p* < 1** | 1.21 | 1.10 – 1.33 | 0.56 | 15.63 | <.0001 |

Note: Shaded rows indicate statistically significant findings after correction. ADHD= Attention/Deficit-Hyperactivity Disorder; CI= Confidence Interval; OR= Odds Ratio; PRS= Polygenic Risk Scores; SUD= Substance Use Disorder.

| **Table S14. In Referred Youth (n=421): Mixed Modeling of Differences in Clinical Profiles between High, Medium, and Low ADHD Polygenic Risk Groups, Controlling for Age, Sex, and Ancestry** | | | | | | |
| --- | --- | --- | --- | --- | --- | --- |
|  | **High risk vs. low risk** | | **Medium risk vs. low risk** | | **Main Effect of PRS** | |
| **ADHD PRS Threshold** | ***beta*** | ***p*-value** | ***beta*** | ***p*-value** | **Wald X^2^(2)** | ***p*-value** |
| ***p* < 1.0 x 10^-6^** | .24 (.09) | .0050^a^ | .13 (.08) | .1112 | 7.98 | .0184 |
| ***p* < 1.0 x 10^-5^** | .22 (.09) | .0114^a^ | .09 (.08) | .2618 | 6.50 | .0387 |
| ***p* < 1.0 x 10^-4^** | .21 (.09) | .0137^a^ | .06 (.08) | .4248 | 6.48 | .0391 |
| ***p* < 0.001** | .13 (.09) | .1420 | .11 (.08) | .1853 | 2.61 | .2711 |
| ***p* < 0.01** | .14 (.09) | .1125 | .08 (.08) | .3038 | 2.60 | .2725 |
| ***p* < 0.05** | .01 (.09) | .8814 | .03 (.08) | .7367 | 0.11 | .9464 |
| ***p* < 0.1** | .14 (.09) | .1103 | -.02 (.08) | .8112 | 4.08 | .1300 |
| ***p* < 0.3** | .16 (.09) | .0648 | .08 (.08) | .2967 | 3.44 | .1790 |
| ***p* < 0.5** | .12 (.09) | .1519 | .06 (.08) | .4674 | 2.07 | .3552 |
| ***p* < 1.0** | .14 (.09) | .1070 | .12 (.08) | .1513 | 3.11 | .2111 |
| Note: Shaded rows indicate statistically significant findings for main effect of PRS after correction. ADHD= Attention/Deficit-Hyperactivity Disorder; PRS= Polygenic Risk Scores; ^a^Comparison significant. | | | | | | |
